# Celebrating breakthrough in dental diagnostics: FDA approval of an AI model for diagnosis of periodontal diseases: A correspondence

**DOI:** 10.1002/hsr2.1573

**Published:** 2023-09-18

**Authors:** Ayesha Khan, Khadija J. Khan, Mehreen A. Ghaza, Tirth Dave, Syeda Shahnoor, Abdul M. Khan, Malik O. Oduoye, Wechuli P. Nafula, Samuel C. Ubechu

**Affiliations:** ^1^ Department of Oral and Maxillofacial Surgery Peshawar Dental College Peshawar Pakistan; ^2^ Department of Oral and Maxillofacial Surgery Ayub Dental Section Abbottabad Pakistan; ^3^ Department of Internal Medicine Bukovinian State Medical University Chernivtsi Ukraine; ^4^ Department of Internal Medicine Dow University of Health Sciences Karachi Pakistan; ^5^ Department of Internal Medicine Ayub Medical College Abbottabad Pakistan; ^6^ Department of Research Ahmadu Bello University Zaria Kaduna State Nigeria; ^7^ Department of Research Medical Research Circle Bukavu D.R. Congo; ^8^ Clinical Medicine Jomo Kenyatta University of Agriculture and Technology Nairobi Kenya; ^9^ Department of Social and Behavioral Sciences University of Nigeria (UNN) Nsukka Enugu State Nigeria; ^10^ Department of Social and Behavioral Sciences Yale University New Haven Connecticut USA

**Keywords:** artificial intelligence, dental diagnostics, dental radiographs, dentistry, periodontal disease

## Abstract

Periodontal diseases are prevalent and have significant implications for oral health and overall well‐being. Current diagnostic methods have limitations in accuracy and standardization. The recent Food and Drug Administration approval of Videa Perio Assist (VPA), an AI model for diagnosing periodontal diseases, presents a breakthrough in dental diagnostics. VPA is a cloud‐based, AI‐powered software that automatically measures and visualizes bone levels associated with each tooth from radiographic images. Clinical testing has demonstrated VPA's efficacy in accurately diagnosing periodontal diseases with high sensitivity and specificity. The integration of AI in dentistry has the potential to revolutionize periodontal disease diagnosis, improve patient care, and enhance decision‐making. However, further research, education, cost‐effectiveness, and collaboration are essential for maximizing the benefits of AI in dental settings. The approval and implementation of VPA mark a significant advancement in dental diagnostics, paving the way for more effective solutions and a healthier global population.

1

Periodontal disease is a chronic inflammatory condition characterized by the progressive degradation of the gums and the surrounding connecting tissues. It affects a staggering 47.2% of adults and is acknowledged as a prominent etiological agent in tooth loss.^1^ The principal cause of periodontal disease is the buildup of bacterial biofilm, known as dental plaque, on the tooth surfaces, which elicits an immune‐mediated inflammatory reaction by the body, leading to the progression of the disease.^2^ The progression of periodontal disease encompasses a continuum of stages, namely gingivitis, early periodontitis, moderate periodontitis, and advanced periodontitis, with each successive stage indicating a deterioration marked by increasing levels of inflammation, deleterious effects on the gingival tissues, progressive bone resorption, and heightened tooth mobility.^2^ Periodontal diseases have also been linked to various systemic conditions, including cardiovascular diseases, diabetes, respiratory conditions, and pregnancy‐related complications.^3^

Considering the significant implications of the disease careful and timely diagnosis is paramount in preventing and improving the treatment outcomes of the disease. Various diagnostic methods are employed for comprehensive periodontal evaluation to assess and diagnose periodontal diseases, including periodontal probing, clinical examination of the gum tissues, assessment of dental X‐rays, analysis of periodontal pocket depth, evaluation of biofilm index, tooth mobility and examination of bleeding on probing, all of which aid in determining the presence, severity, and extent of the disease.^4^ While all these diagnostic methods play a valuable role in identifying periodontal diseases, they each have inherent limitations. For instance, dental radiographs may not effectively visualize soft tissues. Pocket depth measurements and periodontal probing are subject to limitations due to factors such as gingival swelling and anatomical variations, which can impact the accuracy of readings and potentially lead to misinterpretation or incomplete assessment of the periodontal condition. Additionally, these methods rely on subjective assessments, introducing variability in the diagnosis. Recognizing the need to overcome these limitations, there has been a growing interest in exploring the integration of AI models in dentistry. AI holds promise in providing more standardized and accurate results, potentially revolutionizing the field of periodontal disease diagnosis. We write this correspondence to celebrate the Food and Drug Administration (FDA) approval of a novel AI model, Videa Perio Assist (VPA), for diagnosis of periodontal diseases and discussing its role in improving accuracy, standardization, and diagnosis of patients with periodontal diseases.

In dentistry, artificial intelligence is creating a revolution in all sections from the collection of data, creating algorithms for orthodontic procedures, diagnostic records in the aspect of radiographic data, three‐dimensional scans, and cone beam computed tomography for restorative, prosthetics, and digital smile design purposes. Similarly, continuous research is being done in the field of periodontics. Multiple studies have demonstrated the superiority of AI models in diagnosing periodontal diseases. According to a systematic review; these models achieved accuracies between 47% and 81% in diagnosing periodontal disease from intraoral photographs and 73.4% to 99% accuracy in detecting alveolar bone loss from radiographic images.^5^

The recent development in dentistry is the FDA's approval of VPA on February 6, 2023.^6^ VPA is an AI‐assisted measurement tool for diagnosing periodontal diseases. It is a cloud‐based, AI‐powered semi‐automated image processing software device designed to automatically measure and visualize mesial and distal bone levels associated with each tooth from bitewing and periapical radiographs. The measurements can be obtained as linear distances expressed in millimeters or relative percentages of the root length. Using VPA, users can upload radiographs within their image‐viewer and review the results. The device output will show all applicable measurements from one radiograph regardless of the number of teeth present.^6^ The VPA has demonstrated its safety and effectiveness in patients aged 12 years and above. In contrast, the only other FDA‐approved device, Overjet Dental Assist, is only designed for adults aged 22 years and older.

During the clinical testing phase, a total of 189 radiographs and over 2350 lines were examined. VPA demonstrated its compliance with predetermined acceptance criteria on bitewing radiographs, achieving a sensitivity of 92.8%, specificity of 89.4%, and maintaining mean absolute error within the acceptable range. Similarly, VPA met the established acceptance criteria on periapical radiographs, exhibiting a sensitivity of 88.3%, specificity of 87.0%, and maintaining mean absolute error within the defined thresholds. The clinical testing provided compelling evidence of the efficacy of VPA in accurately diagnosing periodontal diseases.^6^

In conclusion, the FDA approval of VPA is a breakthrough in the field of dentistry. The algorithms of this machine would help in making an early and accurate diagnosis, and improve patient care. AI aids in time‐sensitive, critical decisions and eliminates human errors, ensuring superior healthcare quality. Models ensure unbiased radiographic analysis, boosting diagnostic precision by mitigating fatigue‐related variability. AI's efficient processing facilitates timely interventions. Notably, its pattern recognition reveals subtle indicators like bone level changes, missed by humans. This early detection aids preventive measures against periodontal disease progression. However, AI is still in the early stages and to fully understand its use, potential, and limitations; focused education on the matter is essential. VPA should be made cost‐effective and readily available in healthcare settings to facilitate the accurate diagnosis of periodontal diseases. Continued research should be conducted by researchers to further investigate the role of AI in dental settings. Regular monitoring and evaluation of the performance and outcomes of AI models in clinical practice should be done. Most importantly, collaboration between dental professionals, researchers, and AI developers should be encouraged to facilitate further advancements. By integrating such technology into clinical practice, we can expect to have more effective solutions that would lead to a healthier global population in the future.

## AUTHOR CONTRIBUTIONS

**Ayesha Khan**: Conceptualization; data curation; writing—original draft; writing—review and editing. **Khadija J. Khan**: Conceptualization; writing—original draft; writing—review and editing. **Mehreen A. Ghaza**: Writing—original draft; writing—review and editing. **Tirth Dave**: Supervision; validation; writing—original draft; writing—review and editing. **Syeda Shahnoor**: Writing—original draft; writing—review and editing. **Abdul M. Khan**: Writing—original draft; writing—review and editing. **Malik O. Oduoye**: Project administration; supervision; writing—original draft; writing—review and editing. **Wechuli P. Nafula**: Writing—original draft; writing—review and editing. **Samuel C. Ubechu**: Writing—original draft; writing—review and editing.

## CONFLICT OF INTEREST STATEMENT

The authors declare no conflict of interest.

## ETHICS STATEMENT

The authors have nothing to report.

## TRANSPARENCY STATEMENT

The corresponding author Tirth Dave affirms that this manuscript is an honest, accurate, and transparent account of the study being reported; that no important aspects of the study have been omitted; and that any discrepancies from the study as planned (and, if relevant, registered) have been explained.

## Data Availability

The authors have nothing to report.
